# Cumulative Delivery Rate after Providing Full Reimbursement In Vitro Fertilization Programme: A 6-Years Survey

**DOI:** 10.1155/2014/850478

**Published:** 2014-03-09

**Authors:** Urban Vrtacnik, Eda Vrtacnik Bokal, Rok Devjak

**Affiliations:** ^1^Law Firm Miro Senica and Attorneys, Ltd., Barjanska cesta 3, 1000 Ljubljana, Slovenia; ^2^Reproductive Unit, Department of Obstetrics and Gynecology, University Medical Centre Ljubljana, Šlajmerjeva 3, 1000 Ljubljana, Slovenia

## Abstract

Since 1983, Slovenia has been offering well-established, successful, and fully reimbursed IVF programme to infertile couples. On the grounds of data gathered at the Slovenian IVF units we aimed to determine whether the fully accessible IVF treatment system can provide notable success considering cumulative delivery rate (cDR). Longitudinal analysis of getting cDR was performed in 810 IVF cycles of 395 couples who for the first time attended the IVF programme in year 2006 and were followed until year 2012. We calculated the actual and the optimistic cDR. In women aged <38 years the actual cDR was 54% and optimistic DR was 83%, respectively. In women aged ≥38 years the actual cDR was 24 % and optimistic cDR was 27%. These results enable us to report that prospects of the treatment for the women aged <38 years, if they undergo all 6 available IVF cycles, are very positive and quite comparable to the chances of spontaneous conception. Even in older patients it is beneficial to repeat the IVF procedures. Therefore we consider the existing infertility treatment system in Slovenia as an example of good medical practice with high level of beneficence offered to the patients.

## 1. Introduction

Since 1983, Slovenia has been offering well-established and successful IVF treatment programmes to infertile couples. According to Europe IVF monitoring (EIM), Slovenia achieves high rankings in terms of IVF treatment accessibility (number of IVF cycles, ICSI cycles, and frozen-thawed cycles per million inhabitants), while in 2009, the percentage of IVF-related deliveries (4,5%) was even amongst the highest percentages in Europe [1].

In Europe the IVF treatment is performed 2.5 times more frequently compared to the United States [1, 2]. This difference is probably due to different funding systems [3].

In Slovenia, the IVF treatment programmes offer full access to infertile couples. Under the term “full access,” Slovenian medical assurance system recognises (under compulsory and supplementary health insurance) the broadest concept of this term, offering the treatment notwithstanding the treatment prognosis, including full reimbursement of costs of IVF cycles by the state, and allowing reinclusion into the programme after delivery. The limitations of treatment are set only in terms of female age (up to 43 years) and the number of cycles (6 cycles for the first delivery and additional 4 cycles on reinclusion into the programme after the first delivery [4]. These limitations reasonably draw a line between effective and ineffective (futile) treatment, since it has been demonstrated that delivery rate (DR) decreases to the level of futile assistance after the female age of 43 years [5, 6].

Based on our own experience, Slovenia introduced new legislation regarding infertility treatment and biomedically assisted procreation in year 2000 [7]. Before adoption in the Parliament, the legislation had been discussed and drafted by a group of infertility and legal experts and has not been supplemented or amended nor challenged by any person before the Constitutional Court of the Republic of Slovenia since its adoption in year 2000. Together with continued and successful practice of fully reimbursed IVF treatment, this shows efficiency as well as stability of the regulation in question.

In other European countries there are different approaches to IVF treatment in terms of financial access: the policies regarding the level of coverage and the number of cycles covered by the state differ significantly. Some of the countries with full coverage of IVF treatment include Belgium (6 cycles), France (4 cycles), The Netherlands (3 cycles), and Sweden (various number of cycles). “Full coverage” in this context is defined as 100% coverage of at least one cycle of IVF on a national basis. According to Berg Brigham et al. [8], France is supposed to be the only European country where a treatment cycle constitutes a fresh stimulated cycle, while any subsequent transfer using frozen embryos is not considered as a new cycle. We may add, however, that the same policy is also applied in Slovenia.

Since the European Parliament called on member states to ensure the right of couples to universal access to infertility treatment [9] and since the European Society of Human Reproduction and Embryology (ESHRE) concluded that reimbursement policies can have a significant impact on the accessibility and use of ART treatments [3, 10–14], we consider the existing infertility treatment system in Slovenia with the above mentioned characteristics of “full access” as an example of good medical practice which enables the infertile couples to repeat the IVF cycles and in this way to improve the cumulative delivery rate (cDR).

The most often performed analysis to determine the success rates (of IVF clinics or rates on the national level) includes cross-sectional statistics. Also the national reports to the European IVF Monitoring (EIM) at ESHRE are prepared in terms of pregnancy outcome per started cycle or embryo transfer [1]. However, such statistics have only a limited value for individual patients. The DR per each cycle is not very indicative in terms of overall success prognosis of each couple. Couples are usually more interested in their realistic overall prospects on the treatment and their actual chances to conceive a child, if they undergo all IVF cycles. At the same time it is also important for the state to evaluate actual outcomes of its accessible IVF treatment system with full reimbursement, acknowledging all accessible cycles. It may be concluded therefore that better evaluation of IVF treatment should be done by performing longitudinal analysis including the data on repeating IVF cycles.

Therefore the aim of our longitudinal study was to determine cDRs by repeating IVF cycles in infertile couples included into our programmes, also showing cDRs per specific female age groups and different ovarian responses to hormonal stimulation. Additionally we also aimed to critically discuss gathered cDR data in relation to the basic principles of Slovenian IVF treatment system, trying to determine the level of beneficence of current regulation.

## 2. Materials and Methods

### 2.1. Infertile Couples and IVF Cycles

In this longitudinal study we analysed 810 IVF cycles in 395 couples who for the first time attended the IVF program of IVF Unit of Department of Obstetrics and Gynaecology, University Medical Centre Ljubljana, in year 2006 and were followed until year 2012. The repeated IVF cycles were performed at infertility centres in Ljubljana, Maribor, and Postojna. We followed all patients until the end of year 2012 since we expected that in this time period all patients would have completed all 6 available IVF cycles. We aimed to find out whether the fully accessible IVF treatment system in our country can deliver notable success considering cDR for both the individual couple and the state considering the female age and ovarian response to the hormonal stimulation. The patients were divided according to their age (women aged <38 years and women aged ≥38 years) and according to the response to ovarian stimulation (poor: ≤3 retrieved oocytes and normal: >3 retrieved oocytes). Poor prognosis is known to be related to the increasing female age [5, 6] and poor ovarian response to ovarian stimulation by gonadotropins [15–17].

### 2.2. Indications of Infertility

In the observed study population, the main indications for IVF treatment were tubal factor infertility, endocrine factors of infertility, unexplained infertility, endometriosis, male factor infertility, or combinations of these.

### 2.3. Ovarian Hormonal Stimulation

For ovarian stimulation the combination of gonadotropins and GnRH agonist was used preferentially. Controlled ovarian stimulation (COH) was performed with either recombinant follitropin alfa or beta (rFSH) or urinary HMG. The starting dose was administered individually. For oocyte maturation HCG was administered and oocyte retrieval was performed 36 h after HCG administration. At most two embryos were transferred. All supernumerous embryos were cryopreserved. For luteal supplementation progesterone intravaginally was used.

### 2.4. Longitudinal Analysis, Cumulative Pregnancy, and Delivery Rates

We calculated the cumulative pregnancy rate (cPR) and cumulative delivery rate (cDR) for all couples undergoing the first IVF treatment in year 2006 and all their subsequent repeated fresh or frozen-thawed cycles until the end of year 2012. Such follow-up period was considered to be sufficient for all patients to undergo 6 available cycles during this time period. To estimate cPR and cDR we included the repeated IVF cycles of these couples at our IVF unit or other Slovenian IVF units, at the University Medical Centre in Maribor or at the IVF Unit of Gynaecological Hospital in Postojna.

In this longitudinal analysis we wanted to identify the benefit of repeating IVF cycles in terms of achieving better cDR. We also wanted to evaluate the importance of repeating cycles in patients with poor prognosis [5, 6, 15–17].

The women were divided according to age (<38 and ≥38 years) and according to the response to ovarian stimulation (≤3 retrieved oocytes and >3 retrieved oocytes).

The cPRs and cDRs were determined by two approaches, as actual cPR/cDR and as optimistic cPR/cDR. Actual cPRs/cDRs were calculated as cumulative numbers of pregnancies and deliveries in observed group of women without any methodological modification in relation to dropout patients. We further calculated the optimistic cDR, since in the actual study population we acknowledged that certain dropout of the patients occurred irrespective of the fully reimbursed system being available in Slovenia. In optimistic cPR/cDR a statistical correction according to the dropout was performed. A correction assumes that women who did not return for subsequent IVF cycles would have the same chance of delivery as those who remain in the treatment.

### 2.5. Statistical Analysis

Statistical analysis was performed using SPSS for Windows, version 13.0 (SPSS, Inc., Chicago, IL, USA). Student's *t*-test was performed for comparisons of PRs/DRs between the groups. Differences were considered significant when *P* values were < 0.05. For actual cPR and cDR we used Kaplan-Meier method. For optimistic cPR and cDR we used modified Kaplan-Meier method which censors data for women who do not return for further IVF cycles to estimate the cPR/cDR of dropout patients with the same average chance of pregnancy/delivery as for those who returned for treatment.

## 3. Results

Our cohort included 395 women who attended 810 IVF cycles undergoing the first IVF treatment in year 2006 and all their subsequent repeated fresh or frozen-thawed cycles until the end of year 2012.

In their first IVF cycle women had the highest DRs regardless of the age; with each consecutive cycle the DRs were dropping. In the first cycle the DR was 28%, in the second 22%, and in the third 20%. In women under 38 years, a degree of DR was observed in each of six IVF cycles, while in women after 38 years there was no delivery after the second IVF cycle. DRs by women's age according to consecutive number of treatment cycle are presented in Table 1 (see Table 1).

**Figure 1:**
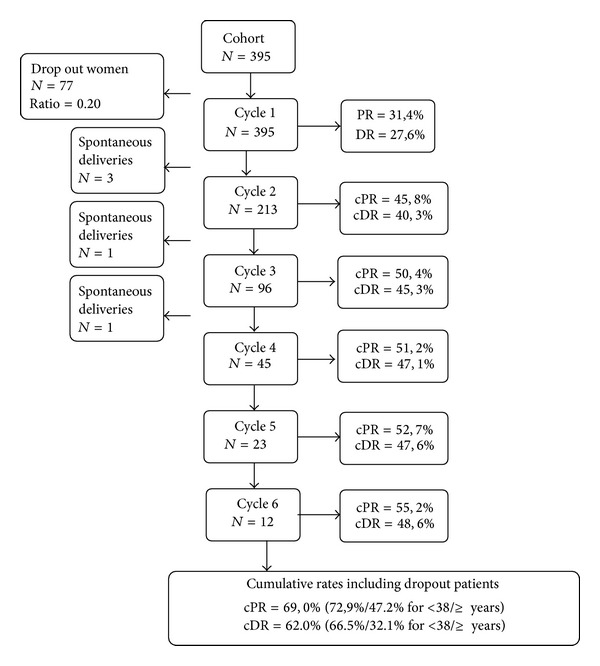
A flowchart of the total cohort of women who started the first IVF cycle in 2006 with subsequent repeated cycles including frozen-thawed cycles and the discontinuation rate (dropout patients). The middle rectangles represent six cycles and number of women participating in each of the cycle. The cPR and cDR are calculated for each cycle in the right rectangles. Dropout and spontaneous deliveries are presented in left rectangles. The actual cPR and cDR according to the age are presented in the bottom rectangle. Legend: cPR: cumulative pregnancy rate; cDR: cumulative delivery rate.

In the longitudinal follow-up analysis, 395 women who started their first IVF cycle in year 2006 were taken into consideration. All first and subsequent fresh and corresponding frozen-thawed cycles of these women performed from year 2006 to the end of year 2012 were analysed. This analysis provided actual cPRs and cDRs for nearly 6 years of follow-up. The cPRs and cDRs, including fresh and thawed cycles, are shown in Figure 1.

The dropout of the patients was 20% (*n* = 77). In the group of women aged <38 years it was 19% (*n* = 60) and in the group of women aged ≥38 years it was 24% (*n* = 17).

The dropout patients are acknowledged as patients without a delivery in the first cycle and not returning for any further cycle. If those women were not included into the analysis, total cDR would have risen to 62% (66.5% in women aged <38 years versus 32.1% in women aged ≥38 years, resp.). Such exclusion was avoided by estimating the optimistic cDRs, statistically supplementing dropout patients with average actual cDR of the programme (see Figure 1).

Actual cPRs and cDRs were calculated according to women's age and ovarian response (see Table 2). In the group of low-responding women after the first IVF cycle there was statistically important difference in DR between women aged <38 years and women aged ≥38 years (0.3 versus 0.09; *P* = 0.018) as well as in actual cDR (0.50 versus 0.19) after available 6 IVF cycles (*P* = 0.003).

In the observed time period the average number of performed cycles per women was 1.91 in the group of women aged <38 years and 2.25 in the group of women aged ≥38 years.

We also calculated the optimistic cDR according to the women's age which assumes that women not repeating IVF cycles would have had the same chance to conceive as those women who maintain in the repeating system for 6 IVF cycles.

**Figure 2:**
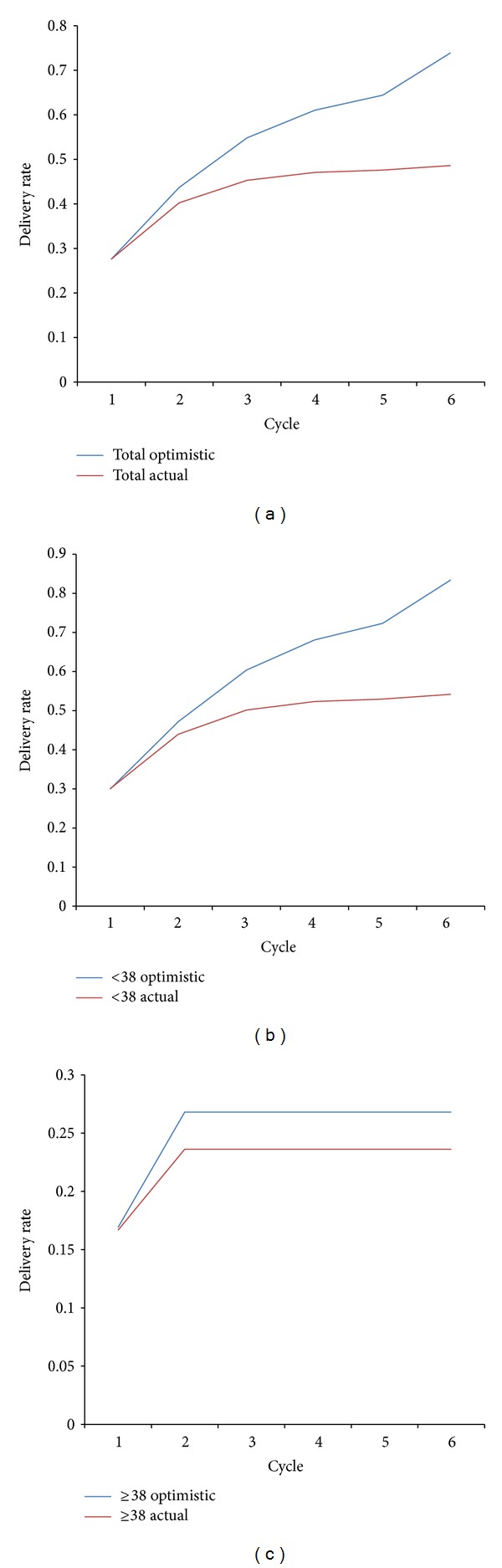
Actual and optimistic realistic cumulative delivery rate (cDR) in cohort (a), women aged <38 years (b), and women aged **≥**38 years (c). Legend: cDR: cumulative delivery rate.

Among the women aged <38 years, the optimistic cDR after 6 IVF cycles was 83%. Among the women aged ≥38 years, the optimistic cDR after 6 IVF cycles was only 27%. The difference between optimistic and realistic cDR in younger women (<38 years) was 29% but in older women (≥38 years) was only 3% (Figure 2).

## 4. Discussion

The results of this study showed that in younger women (<38 years) the real cDR was 54% and optimistic cDR was 83%, respectively. In older women (≥38 years) the real cDR was 24% and optimistic cDR was 27%. These results enable us to report that prospects of the treatment in younger women, if they undergo all 6 available IVF cycles, are very high and quite comparable to the spontaneous conception. Even in older women it appears to be beneficent to repeat IVF cycles. This confirms the importance of repeated IVF cycles in assisted procreation and consequentially the importance of full coverage reimbursement IVF programmes.

In European countries, the IVF birth rate has been estimated to represent between 1% and 4.5% of all deliveries. In Slovenia birth rate reaches 4.5% of all deliveries; in the last European IVF Monitoring (EIM) report for the year 2009 it was the highest in Europe, besides Denmark, one of the leading European countries in the ART programme [1]. This can be achieved within fully reimbursed IVF systems which allow patients to repeat the IVF cycles and improve cDRs (as shown above). We consider such data to be important for the value and quality of the IVF programme in the country.

Recently, the usual annual cross-sectional analysis of PRs and DRs per started cycle and per embryo transfer was performed by the EIM which has collected the clinical data of most of European countries (IVF centres), respectively [1]. Despite being important, these data do not provide the information on the potential outcome and success of treatment for the individual couple or patient, since they do not show the overall success in repeating IVF cycles. Therefore the longitudinal analysis to provide the cDRs seems to be of greater importance.

Therefore we primarily reported the actual cDR in the IVF programme using longitudinal analysis. We were able to do this with accuracy, since there are only three IVF centres in Slovenia. We were able to gather data from all IVF centres in the country and thus avoid bias problems related to “moving patients” within centres. We believe that, so far, the present analysis may be the first one revealing data on the IVF treatment in the whole country and not only data related to individual IVF centre [18–20].

Additionally, with the statistical optimistic assumption we have the opportunity to report to the couples/patients their optimal expectation, if they undergo all 6 cycles being available within the programme. Nevertheless, the figures on optimistic cDRs may be overestimated and must be interpreted with a caution [18–30]. Therefore both real actual values and optimistic estimations must be considered simultaneously.

The actual cDR for the whole cohort of patients was 49% while the optimistic estimate of the DR, which assumed that patients, who did not repeat IVF cycles, had the same chance of a delivery, reached 74%. This is in agreement with the Boston IVF centre achieving cDR between 51% and 72% performing conservative and optimistic calculation [27]. This comparison is feasible because of similar insurance policy for achieving the first delivery in both environments; in Massachusetts, the insurance benefits of up to six IVF cycles were offered to many patients [27].

Like several other investigators, we found that patients who did not return for further IVF cycles had worse chances to conceive than those who returned and continued with treatment [18, 19, 21, 25, 27].

It is known that fertility competence declines with increased female age in both the general population [18] as well as in subfertile women [31]. In our study, in younger women (<38 years) the repetition of IVF cycles resulted in a similar cDR in normally responding and in low-responding patients (54.8% and 50.0%, resp.). By estimating optimistic cDR which assumes that women who did not return for subsequent IVF cycles had the same chance of delivery as those who remained in the treatment, we reached the value of 83% cDR in younger women (<38 years). This result enables us to report to the younger women that their optimal expectation, if they repeat all 6 IVF cycles, comes very high and seems to be comparable to the spontaneous conception [18, 32].

On the other hand, the situation is different in older women (≥38 years). In normal-responding older women cDR was 28% and in low-responding women was 19%, which brings an average of 24%. All deliveries were achieved in the first or second IVF cycle. From the literature it is known that the overall live birth rate per started cycle for women who initiated an ART cycle at age 40 years or above is 9.7% and cumulative delivery rate is 28% [20]. It is interesting that 87% of women in this group who achieved delivery conceived a child within the first three IVF cycles [20]. This is also in accordance with the results of our study; however we believe that larger number of older patients would have to be included in the study (sustaining the treatment after the third cycle) and meta-analysis should be performed. This may be achievable in cross-border studies. According to the data from the literature and our own data the age limit of 43 years is reliable, since assisted reproductive technology has a reasonable, beyond futile chance of success (>5% delivery rate) until the age of forty-three years [20].

If IVF treatment in Slovenia had not been fully reimbursed (6 cycles and additional 4 cycles after delivery), we would have not achieved such success in repeated IVF cycles. From the literature it is known that relatively large number of clinical pregnancies is achieved by repeated IVF attempts [33]. We strongly believe that, in the case of payable IVF cycles, these children would not have been conceived, since infertile patients usually do not sustain expensive IVF treatments [34]. Such situation was experienced in Germany after the German healthcare modernisation law had been implemented in year 2004, whereas the new legislation introduced the 50% copayment of the total ART treatment instead of the full reimbursement provided by the state. This led to a drastic reduction in ART treatment cycles per year, from 105,576 cycles in year 2003 to 61,950 cycles in year 2004 and 59,117 cycles in year 2005 [35], and showed that the lack of reimbursement can act as a barrier to people using ART and decrease a number of children born in the IVF programme [36, 37].

## 5. Conclusion

Our results show that by repeating IVF cycles actual cDR was 49% while optimistic (estimated) cDR was 74%. In younger women (<38 years), the actual cDR was 54% and optimistic cDR was 83%, while in older women (≥38 years) the actual cDR was 24% and optimistic cDR was 27%. Considering high cDRs in younger women, which are close to natural conception rates, we encourage early and sustained IVF treatment.

On the basis of overall reported actual and optimistic cDRs we may conclude that broad inclusion of patients (only limited by age) and full reimbursement of costs of IVF treatment by the state for 6 consecutive cycles and 4 consecutive cycles after a delivery are proven to be beneficent for the couples. No such cDRs would have been achieved if such system had not been established, since many patients do not repeat cycles if they are obliged to contribute financially to the treatment [35, 36]. Even the dropout which is not cost-related (difference between actual and optimistic DRs, 29% in younger women (<38 years) and 3% in older women (≥38 years)) shows the importance of repeated cycles.

From the legal perspective, we may observe, however, that these elements have not been fully instrumentally implemented into the Slovenian legislation. Namely, Infertility Treatment and Procedures of Biomedically Assisted Procreation Act [7] covers only the basic principles of IVF treatment whereas the specifics (including age limit and number of cycles) are left to be governed by the regulation of Health Insurance Institute of Slovenia [4] which can be changed by the bodies of the institute under consent of the Ministry of Health if the rights are related to compulsory medical insurance. It has to be also noted that legislation [38] only provides 85% of reimbursement of the infertility treatment (assisted procreation) costs, while full reimbursement of costs is agreed within contractual relations between legal entities acting on behalf of IVF centres and Health Insurance Institute of Slovenia.

Since the right to assisted procreation is considered as a part of the constitutional Freedom of Choice in Childbearing [39, 40] and since only the legislation can determine the manners in which (constitutional) rights and freedoms are exercised [41] we believe that basic elements of the IVF system should be governed by the law. Such conclusion is also confirmed beyond legal argumentation with the findings of this paper on cDRs, showing that repeated reimbursed cycles are crucial in terms of exercising the right to (successful) IVF treatment. In terms of the equality in exercising constitutional Freedom of Choice in Childbearing the successful IVF treatment shall be considered as such only if it can deliver comparable results to the natural conception and thus help to eliminate differences between fertile individuals and those infertile individuals that can be helped by medical assistance in procreation.

## Figures and Tables

**Table 1:** Delivery rates (DRs) by women's age according to consecutive number of treatment IVF cycle.

	Cycle 1	Cycle 2	Cycle 3	Cycle 4	Cycle 5	Cycle 6
DR (*n*)	0.28 (109)	0.22 (50)	0.20 (20)	0.14 (7)	0.09 (2)	0.27 (4)
DR < 38 (*n*)	0.30 (97)	0.25 (45)	0.25 (20)	0.19 (7)	0.13 (2)	0.40 (4)
DR ≥ 38 (*n*)	0.17 (12)	0.12 (5)	0 (0)	0 (0)	0 (0)	0 (0)

**Table 2:** Pregnancy rates and delivery rates of first cycles started in year 2006 and actual cumulative pregnancy rates until year 2012.

			*P* value	Total
Age (years)	<38	≥38		
First cycle in 2006 (*n*)	324	71		395
PR (*n*)	0.33 (106)	0.25 (18)	0.208	0.31 (124)
DR (*n*)	0.30 (97)	0.17 (12)	0.013	0.28 (109)
Normal responders (*n*)	270	40		310
PR (*n*)	0.33 (88)	0.28 (11)	0.521	0.32 (99)
DR (*n*)	0.30 (81)	0.23 (9)	0.305	0.29 (90)
Low responders (*n*)	54	31		85
PR (*n*)	0.33 (18)	0.22 (7)	0.287	0.29 (25)
DR (*n*)	0.30 (16)	0.09 (3)	0.018	0.22 (19)
Cumulative pregnancy and delivery rates until the end of 2012				
Average number of cycles-total	1.91	2.25	0.053	1.97
Average number of cycles-normal/low responders	1.87/2.13	2.02/2.54		1.89/2.28
cPR (*n*)	0.58 (189)	0.35 (25)	<0.001	0.54 (214)
cDR (*n*)	0.54 (175)	0.24 (17)	<0.001	0.49 (192)
Normal responders (*n*)	270	40		310
cPR (*n*)	0.59 (158)	0.35 (14)	0.005	0.55 (172)
cDR (*n*)	0.55 (148)	0.28 (11)	0.001	0.51 (159)
Low responders (*n*)	54	31		85
cPR (*n*)	0.57 (31)	0.36 (11)	0.052	0.49 (42)
cDR (*n*)	0.50 (27)	0.19 (6)	0.003	0.39 (33)

Legend: PR: pregnancy rate; DR: delivery rate; cPR: cumulative pregnancy rate, and cDR: cumulative delivery rate.
